# Cancer immunotherapy strategies that target the cGAS-STING pathway

**DOI:** 10.3389/fimmu.2022.996663

**Published:** 2022-10-24

**Authors:** Zhuoying Tian, Yue Zeng, Yurong Peng, Junqi Liu, Fang Wu

**Affiliations:** ^1^ Department of Oncology, The Second Xiangya Hospital, Central South University, Changsha, China; ^2^ Xiangya School of Medicine, Central South University, Changsha, China; ^3^ Hunan Cancer Mega-Data Intelligent Application and Engineering Research Centre, Changsha, China; ^4^ Hunan Key Laboratory of Tumor Models and Individualized Medicine, The Second Xiangya Hospital, Central South University, Changsha, China; ^5^ Hunan Key Laboratory of Early Diagnosis and Precision Therapy in Lung Cancer, The Second Xiangya Hospital, Central South University, Changsha, China

**Keywords:** cGAS, STING, cancer-immunity cycle, immunotherapy, tumor

## Abstract

Activation of the cGAS-STING pathway by cytoplasmic DNA induces the production of Type-1 interferons. Recent advances in research suggest that the cGAS-STING pathway is involved in different parts of the cancer-immunity cycle (CIC) to promote or suppress antitumor immune responses. Combination therapy of STING agonists has made certain progress in preclinical as well as clinical trials, but the selection of combination therapy regimens remains a challenge. In this review, we summarize the role of the cGAS-STING in all aspects of CIC, and focus on the combination immunotherapy strategies of STING agonists and current unsolved challenges.

## 1 Introduction

Cyclic GMP-AMP (cGAMP) synthase (cGAS) has been identified as a cytoplasmic double-stranded DNA sensor that plays a key role in Type-1 interferon and inflammatory responses *via* a Stimulator of Interferon Genes (STING)-dependent signaling pathway (1). This pathway has been demonstrated to have a regulatory role in metabolic endocrine diseases (2–5), viral infections (6, 7), autoimmune diseases (8, 9), and neurological disorders (10, 11). In recent years, there is increasing evidence that the cGAS-STING pathway is closely related to the occurrence, development and regression of cancer. The cGAS-STING pathway regulates various aspects of the Cancer-Immunity Cycle (CIC), including tumor antigen release (12), antigen presentation (13), the priming and activation of T cells (14), the trafficking and infiltration of T cells into tumor tissues (15), and the recognition and killing of tumor cells by T cells (16). The cGAS-STING pathway plays an anti-tumor or pro-tumor role.

In this review, we summarize the role of the cGAS-STING in all aspects of CIC, and focus on the combination immunotherapy strategies of STING agonists and current unsolved challenges.

## 2 Overview of the cGAS–STING signaling

cGAS is a cytosolic DNA receptor activated by double-stranded DNA (dsDNA) in a sequence-independent but length-dependent manner (1, 17). cGAS catalyzes the conversion of GTP and ATP to 2’3’-Cyclic GMP-AMP (2’3’-cGAMP) (18, 19), which binds to STING and promotes its translocation from the endoplasmic reticulum (ER) to Golgi (20, 21). STING recruits and activates TANK binding kinase-1 (TBK1), which in turn promotes the translocation of interferon regulatory factor 3 (IRF3) into the nucleus where it promotes the production of Type-1 interferon and the transcription of interferon-stimulated genes (ISGs) (22, 23). STING also binds and stimulates IκB kinase (IKK), which mediates the activation of canonical and non-canonical NF-kB pathways (24). After signal transduction is terminated, STING is transferred to endolysosomes for degradation (22).

## 3 The cGAS-STING pathway regulates the cancer-immunity cycle

Mounting evidence has demonstrated that the cGAS-STING pathway plays an important regulatory role in all stages of the cancer-immunity cycle, either activating or suppressing anti-tumor immune responses, depending on the strength and timing of the activation of the cGAS-STING pathway and the type and state of the tumors (14, 25–27).

### 3.1 The cGAS-STING pathway increases tumor antigen release by promoting apoptosis

During normal mitosis, cGAS has a higher affinity for nucleosomes compared to dsDNA, thus preventing cGAS dimerization and activation (1). However, when Taxane drugs interfere with mitosis leading to mitotic arrest, the accumulation of phosphorylated IRF3 which is induced by the cGAS inhibits the expression of the anti-apoptotic protein BCL-xL, triggering apoptosis *via* mitochondrial outer membrane permeabilization (MOMP) (12). In addition, Type-1 interferon and TNFα produced by the cGAS-STING activation can stimulate the expression of the pro-apoptotic molecule, NOXA, in neighboring cells *via* paracrine secretion. This induces apoptotic priming, meaning the cancer cells undergo MOMP propensity (28, 29). Analysis of The Cancer Genome Atlas (TCGA) datasets showed that lung and ovarian cancer patients with high cGAS expression were more sensitive to paclitaxel treatment (12). The 2 ‘ 3 ‘ -cGAMP analogue, c-di-AMP, activates the STING pathway to induce apoptosis in estrogen receptor-negative breast cancer cells, resulting in the release of tumor antigens (TAs) and propagation of the cancer-immunity cycle (30).

### 3.2 The cGAS-STING pathway facilitates the processing and presentation of tumor antigens

Dendritic cells (DCs) are considered to be the main antigen-presenting cells (APCs) responsible for the priming of anti-tumor T cells. Type-1 IFN production promotes DC maturation, upregulates the expression of molecules such as MHCI, MHCII, CD40, CD80, CD86 (13) on the DCs surface (31), and enhances DC migration to tumor draining lymph nodes (TDLNs) migration (32). Although T cell activation occurs mainly in TDLNs, STING signaling has been reported to induce the formation of intra-tumor tertiary lymphoid structures (TLS) in a mouse model of melanoma (33), where DCs may activate T cells, thereby skipping the need for migration to TDLNs (34). In addition, it has been reported that in the tumor microenvironment (TME), cancer cells transfer cGAMP into tumor-associated DCs *via* gap junctions, leading to the activation of pathways downstream of the cGAS-STING (31, 35).

### 3.3 The cGAS-STING pathway has a dichotomous effect on the priming and activation of T cells

Although it is well known that the cGAS-STING pathway plays a key role in the regulation of T cell priming and activation, the strength and timing of the activation of this signaling pathway may have opposing effects (14).

Moderate activation of the cGAS-STING pathway upregulates the expression of the TA-MHC I complex on the cell surface of DCs, which is recognized by TCRs, leading to the activation of CD8+ cytotoxic T cells (CTLs) (31).. Moreover, by increasing the expression of the transcription factor TCF1, the cGAS-STING pathway-mediated Type-1 interferon increases the activity of stem-like CD8+ T cells (36), which are capable of self-renewal, persistence, and differentiation potential (37–39). It has been reported that the cGAS agonist Manganese (40), low-dose STING agonists ADU-S100 (S100) (14), Vadimezan (DMXAA) (41), and STINGV155M (a constitutively activating mutation of STING) (42) all have the ability to enhance the activity of CTLs thereby producing durable antitumor immunity. Consistent with these findings, STING-deficiency reduces CD8+ T cell activity in mice (43).

However, high doses of ADU-S100 lead to substantial T cell death and impaired antitumor immunity (14). This may be attributed to the activation of the non-type I IFN domain of STING that disrupts calcium homeostasis, thereby stimulating T cells to be highly responsive to TCR signaling-induced endoplasmic reticulum stress, leading to T cell death (26, 27).

### 3.4 Activation of the cGAS-STING pathway promotes the trafficking and infiltration of T cells

CTLs need to leave TDLNs and enter the tumor tissue *via* blood vessels in order to recognize and kill cancer cells (44). The cGAS-STING pathway-induced Type-1 interferon response drives the expression of multiple chemokines such as CXCL9, CXCL10, and CCL5, that act as chemical gradients to direct CTLs into the tumor tissue (45–47). IFN I signaling also increases the expression of E selectin, VCAM-1, and ICAM-1 in endothelial cells, enhances vascular permeability, and facilitates immune cell extravasation, thus enhancing the antitumor effect (15).

The tumor vasculature is disorganized and immature, with loose connections and low pericyte coverage. In addition, this vascular system does not provide a continuous blood supply to the tumor tissue, thus increasing the distant metastasis of tumor cells and decreasing the tropism of CTLs to TME (48–51). The cGAS-STING pathway-induced activation of Type-1 interferon upregulates the vascular normalization genes such as Cdh5, Angpt1, Pdgfrb, Mcam, and Col4a. These genes induce the normalization of tumor vasculature with increased pericyte coverage and more intact basement membrane, facilitating infiltration of CTLs into tumor tissue (33, 52, 53). Consistent with these findings, STING deficiency reduces the expression of these genes (53). However, vascular endothelial growth factor (VEGF)/VEGFR2 can negatively regulate Type-1 interferon signaling through ubiquitin-mediated IFNAR degradation, leading to the inhibition of Type-1 interferon action in VEGF-rich tumor tissues (53). Combining STING agonists with VEGFR2 blockers not only attenuates the negative effects of VEGF, but also synergistically promotes tumor vascular normalization (53).

### 3.5 The cGAS-STING pathway has a dichotomous effect on the recognition and killing of cancer cells by T cells

Activation of the cGAS-STING pathway not only induces CTLs-mediated cancer cell death by upregulating MHC-I expression on the surface of cancer cells (54), but also activates NK cells to kill tumor cells, especially those with reduced or absent MHC-I expression (55–58). In addition, in the tumor microenvironment (TME), tumor derived cGAMP can be transferred from tumor cells to immune cells to trigger the STING pathway in immune cells and activate the antitumor response of NK cells (59). The death of cancer cells induces the release of tumor antigens, leading to the initiation of a new round of CIC.

The programmed cell death protein 1 (PD-1) expressed by T cells binds to the ligand PD-L1 on the surface of tumor cells, which inhibits the clearance of tumor cells by effector T cells (60–63). Activation of the cGAS-STING pathway has been demonstrated to increase the expression of PD-L1 on the surface of tumor cells and thus attenuate the activity of CTLs, which has been confirmed in models of liver cancer (64), melanoma (65), non-small cell lung cancer (NSCLC) (16) and small cell lung cancer (SCLC) (46, 66). The antitumor effects of STING agonists were enhanced when combined with PD-L1 or PD-1 blockers (40, 67, 68).

It has been found that activation of the cGAS-STING pathway may induce the formation of immunosuppressive TMEs and negatively regulate the killing effect of CTLs (69, 70). (IDO1) is an enzyme that catalyzes tryptophan into kynurenine, which inhibits the proliferation of T cells and promotes the differentiation of Tregs and the infiltration of myeloid-derived suppressor cells (MDSCs) (71, 72). Activation of the cGAS-STING pathway increases IDO1 expression (73), which has been validated in colorectal cancer (74, 75). Analysis of the TCGA dataset revealed that infiltration of Tregs and MDSCs positively correlated with STING expression in pancreatic cancer, bladder urothelial carcinoma, breast cancer, liver cancer, prostate adenocarcinoma, and thyroid cancer (76). Interestingly, Eslam Mohamed et al. (77) proposed that PERK-deficient MDSCs lead to activation of their own STING signaling, reprogramming immunosuppressed MDSCs into myeloid cells that activate CD8+ T cell-mediated anticancer immunity.

In addition, DNA damage-mediated activation of the cGAS-independent non-canonical STING signaling primarily activates NF-kB and promotes IL-6 production, which is associated with pro-tumor response (78–80). 2’3’ -cGAMP transferred from tumor cells to astrocytes activates NF-κB signaling, thereby promoting brain metastasis and chemoresistance (81). Since TBK1 and STING inhibitors do not block non-canonical STING, NF-kB inhibitors may be an option to reduce the pro-tumorigenic response (79).

## 4 The mechanism underlying the inhibition of the cGAS-STING pathway in tumor

An increasing number of investigations have indicated that the activity of the cGAS-STING pathway is inhibited in several tumors due to the regulation of multiple mechanisms. Mutant p53 inhibits the activation of the cGAS-STING-TBK1-IRF3 pathway and promotes tumor progression by interacting with and inhibiting TBK1 activity (82). Mutant NF2 is induced by activated IRF3 to form cellular condensates, which inhibit TBK1 activity, particularly in human vestibular nerve sheath tumors (83). As a hydrolase of cGAMP, ecto-nucleotide pyrophosphatases 1 (ENPP1) impedes the antitumor immune response by blocking cGAMP transfer from tumor cells to immune cells to trigger the STING pathway (84). Hypoxia, a feature of solid cancers, upregulates RNASEH2A *via* HIF2α, which may limit activation of the cGAS-STING signaling by reducing nuclear DNA release. Hypoxia is associated with poor prognosis of hepatocellular carcinoma (85). In a mouse model of ovarian cancer, the SETDB1-TRIM28 complex inhibited the formation of micronuclei in the cytoplasm, thereby inhibiting the activity of the cGAS-STING pathway and suppressing anti-tumor immunity (86). TIM-3 may inhibit the activation of the cGAS-STING pathway by suppressing the uptake of extracellular DNA by DCs, which has been demonstrated in breast cancer models (87).

Thus, blocking the mechanism underlying the inhibition of the cGAS-STING pathway may be an option for the treatment of tumors with suppressed activity of the cGAS-STING, though the existing intervention methods remain immature. In contrast, using agonists to activate the cGAS-STING signaling pathway, thereby antagonizing the inhibitory signals of this pathway and reversing the immunosuppressive state, may be a more feasible approach, which is expected to break the resistance bottleneck of these tumor immunotherapies.

## 5 Immune combination therapy of the cGAS-STING

**Figure 1:**
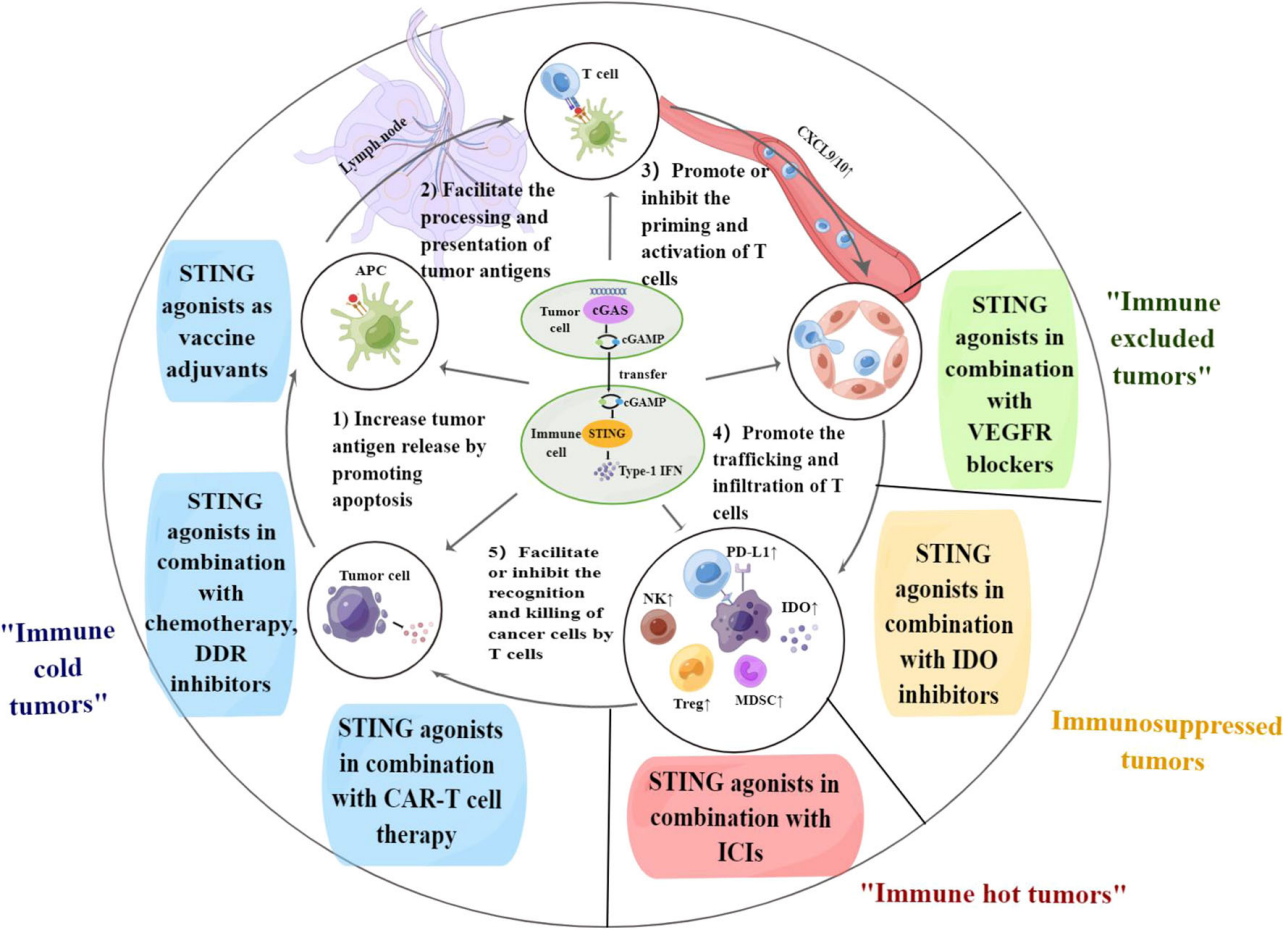
The cGAS-STING pathway regulates each step of the cancer-immunity cycle. Combination therapy of STING agonists can target different steps of the cancer-immunity cycle and contribute to solving immunotherapy challenges in the corresponding immune types of tumors. APC, antigen-presenting cell; PD-L1, programmed death-ligand 1; DDR, DNA damage response; VEGF, vascular endothelial growth factor; IDO, indoleamine 2,3-dioxygenase; ICIs, immune checkpoint inhibitors; CAR-T cell, chimeric antigen receptor-T cell. By Figdraw.

As previously mentioned, the regulation of tumor immunity by the cGAS-STING pathway is dichotomous; therefore, STING agonists applied alone may carry the side effect of immunosuppression. However, combined STING agonists with other suitable antitumor therapies can mechanistically synergize, as demonstrated in clinical and preclinical models (**Figure 1**).

### 5.1 Combination therapy to promote tumor antigen release and presentation

Due to the low mutational burden and low expression of antigen-presentation markers, “immune cold tumors” lack infiltration of CTLs both inside and at the margins of the tumor, which respond poorly to immune checkpoint inhibitors (ICIs) and are often associated with poor prognosis (88–90). Therefore, such combination therapies are essential to overcome the immune deficiency and convert cold tumors into hot tumors.

#### 5.1.1 STING agonists in combination with chemotherapy

STING agonists in combination with chemotherapy have shown promising efficacy in preclinical trials. The combination of cisplatin and cGAMP showed effective CXCR3-dependent antitumor effects in a mouse model of head and neck squamous cell carcinoma (HNSCC) (91). However, several clinical trials of STING agonists in combination with chemotherapy have been completed without achieving expected efficacy. The poor performance of the STING agonist ASA404 in clinical trials may be due to the fact that ASA404 selectively binds to mice, but not to human STING. Therefore, STING agonists with higher affinity for humans need to be rationally designed to enhance antitumor efficacy.

#### 5.1.2 STING agonists in combination with DNA damage response inhibitors

Homologous recombination repair (HRR)-deficient tumors result in a higher tumor mutational load, including KEAP1-mutated non-small cell lung cancer (NSCLC) (92), BRCA1/2-deficient tumors (93, 94), microsatellite instability (MSI) colorectal cancer (CRC) (95), and small cell lung cancer (SCLC) (66) characterized by widespread deletion of two key regulators of the cell cycle checkpoint pathway, TP53 and RB1. Such tumors exhibit sensitivity to DDR inhibitors, and persistent high levels of DNA damage in their cells contribute to activation of the cGAS-STING pathway. It was revealed that combination therapy of DDR inhibitors (including PARP inhibitor olaparib and CHK1 inhibitor prexasertib) and STING agonists demonstrated beneficial therapeutic effects in such tumors, superior to both drugs monotherapy (66, 92–95). Thus, combination therapy with DDR inhibitors and STING agonists is expected to be a promising treatment for HRR-deficient tumors.

#### 5.1.3 STING agonists as vaccine adjuvants

Recently, several studies have demonstrated that STING agonists can serve as adjuvants for tumor vaccines and exert beneficial effects in antitumor therapy. Matteo Rossi et al. (96) discovered that the combination of STING agonists with therapeutic protein vaccines significantly reduced the rate of tumor growth and improved the efficacy of therapeutic vaccination, which was demonstrated in a variety of mouse tumor models. CDGSF, a novel STING agonist that induces a “hot” tumor microenvironment to inhibit melanoma progression, has been shown to induce a robust adaptive immune response as an adjuvant to SARS-CoV-2 stinger protein and has great potential to be an adjuvant for cancer vaccines (97).

### 5.2 STING agonists combined with VEGFR blockers to promote the trafficking and infiltration of T cells

The combination of STING agonists and VEGFR blockers collaboratively drives the infiltration of CTLs into the tumor core, which is essential for “immune excluded tumors”. In immune excluded tumors, CTLs aggregate at the tumor border but cannot invade the tumor interior, possibly due to the lack of T-cell chemokines or abnormal tumor vascular formation barriers (69). Anlotinib, a tyrosine kinase inhibitor (TKI), inhibits tumor angiogenesis by blocking multiple targets such as VEGFR, PDGFR, and FGFR. A recent study revealed that the antitumor effects of anlotinib were also associated with activation of the cGAS-STING pathway, which was confirmed in a mouse model of gastric cancer (98). Another study confirmed that triple immunotherapy with STING agonists, anti-VEGFR2 antibodies, and anti-PD-1 or anti-CTLA-4 antibodies was more potent and durable in mouse models of lung and colon cancer, extending survival in mice resistant to ICIs or anti-angiogenic therapy (53).

### 5.3 Combination therapy to facilitate the recognition and killing of tumor cells by T cells

#### 5.3.1 STING agonists in combination with chimeric antigen receptor -T cell therapy

CAR-T cell therapy is one of the promising anti-cancer therapies that has achieved excellent efficacy in treating hematologic tumors (99), but has a lower success rate in treating patients with solid tumors, which may be due to insufficient infiltration of CAR T cells into tumor tissue, immunosuppression TME-induced functional suppression, and CAR T cell exhaustion (100, 101).

*In situ* mouse mammary tumor model, administration of STING agonists DMXAA or cGAMP at sites distant from the tumor significantly enhanced the efficacy of Th/Tc17 CAR T cells, which may be related to the upregulation of chemokines CXCL9 and CXCL10 by STING agonists to promote the infiltration of CAR T cells into the tumor tissue. Furthermore, sustained tumor regression was only achieved in combination with anti-PD-1 monoclonal antibodies, possibly due to anti-PD-1 antibodies reversing CAR T-cell exhaustion (102). Feng Ji et al. also confirmed that PARPi can activate the cGAS-STING pathway to enhance the efficacy of CD70 CAR-T cells on renal cancer (103).

#### 5.3.2 STING agonists in combination with immune checkpoint inhibitors

“Hot tumors” already contain large numbers of infiltrating T cells that were once activated but are depleted or malfunctioning due to the expression of a range of immunosuppressive receptors, including CTLA4 and PD-1 (69). As mentioned previously, activation of the cGAS-STING pathway promotes the infiltration of CTLs into tumor tissue and upregulates the expression of PD-L1 on the surface of cancer cells. While the therapeutic efficacy of immune checkpoint inhibitors (ICIs) correlates with the baseline infiltration level of CTLs in tumor tissue. Therefore, the combination of STING agonists and ICIs for the treatment of immune hot tumors may synergize.

The combination of STING agonists and ICIs is currently achieving some efficacy in clinical trials. A multicenter Phase 2 clinical trial demonstrated a complete response of 16.7% and a partial response of 83.3% (NCT03937141) when ADU-S100 (a STING agonist) and pembrolizumab were used together in the treatment of recurrent or metastatic head and neck cancer. An open-label phase 1 clinical trial for patients with advanced metastatic solid tumors showed that Mn2+, which can activate cGAS in combination with anti-PD-1 antibodies, has promising efficacy, with an objective response rate of 45.5% and a disease control rate of 90.9% (NCT03991559) (40). In preclinical model of HPV + oral cancer, intratumoral injection of STING agonist combined with systemic treatment with anti-PD-1 antibodies and anti-CTLA-4 antibodies resulted in sustained tumor regression in 71% of mice, significantly higher than the efficacy of PD-1blocker alone (104). In mouse melanoma models with B16F10 and BRAF mutations, the combination use of LP-cGAMP and anti-PD-L1 antibody achieved stronger and more durable efficacy than LP-cGAMP or anti-PD-L1 alone (105).

#### 5.3.3 STING agonists in combination with IDO inhibitors

In immunosuppressed tumors, immune infiltration is present in the tumor lesion, but the degree of infiltration is not high (69). As previously mentioned, while activation of the cGAS-STING pathway promotes immune infiltration, it also upregulates the expression of the immunosuppressive factor IDO. Therefore, combining STING agonists with IDO inhibitors may be a promising option to reverse immunosuppression and promote immunosuppressed tumors to become hot tumors, thereby improving the efficacy of ICIs.

The combination of STING agonist and IDO inhibitor is currently in preclinical. In a mouse colorectal cancer model, the STING agonist diABZI in combination with the IDO inhibitor 1-MT significantly inhibited tumor growth, promoting the recruitment of CTLs and inhibiting the infiltration of MDSCs (75).

## 6 Conclusion and perspectives

The cGAS-STING pathway mediates various aspects of the cancer immune cycle (CIC) to enhance or attenuate anti-tumor immune responses. Combination therapy of STING agonists can target different steps of the cancer-immunity cycle and contribute to solving immunotherapy challenges in the corresponding tumor immune-phenotype. In addition, the activity of the cGAS-STING pathway is inhibited in several tumors due to negative regulation by multiple mechanisms such as TIM-3, ENPP1.

However, the following challenges need to be solved for STING agonists to be clinically applied on a large scale. First, for specific patients, whether STING agonists are immunopromoting or immunosuppressive is unclear and may be related to their tumor type and immune microenvironmental characteristics, which need to be further explored. Second, STING agonists with higher affinity for humans need to be rationally designed to enhance antitumor efficacy. Third, more potential STING agonist combination therapy strategies need to be explored, such as STING agonist in combination with TIM-3 inhibitor, ENPP1 inhibitors.

In summary, we believe that the cGAS-STING pathway manipulation will have a promising future in tumor immunotherapy.

## Author contributions

ZT wrote the manuscript. ZT, YZ, YP, JL and FW revised the paper. All authors contributed to the article and approved the submitted version.

## Funding

This work was supported by: 1) Hunan Provincial Science Fund for Excellent Young Scholars (Grant No. 2021JJ20088); 2) Changsha Municipal Science and Technology Bureau (Grant No. kq1907077).

## Acknowledgments

The authors sincerely thank Dr. Feng Liu providing language help and valuable discussion points. The authors sincerely thank the multidisciplinary team (MDT) of thoracic oncology, the Second Xiangya Hospital, Central South University.

## Conflict of interest

The authors declare that the research was conducted in the absence of any commercial or financial relationships that could be construed as a potential conflict of interest.

## Publisher’s note

All claims expressed in this article are solely those of the authors and do not necessarily represent those of their affiliated organizations, or those of the publisher, the editors and the reviewers. Any product that may be evaluated in this article, or claim that may be made by its manufacturer, is not guaranteed or endorsed by the publisher.
